# Frequency-Domain Analysis and Optimization of Capacitive Angle Sensors

**DOI:** 10.3390/s26185762

**Published:** 2026-09-10

**Authors:** Pei Huang, Changliang Wu, Xiaokang Liu, Xianjie Xiao

**Affiliations:** 1 School of Mechanical Engineering, Beijing Institute of Technology, Beijing 100081, China; 3220195041@bit.edu.cn (P.H.); 3220225054@bit.edu.cn (C.W.); 2 Engineering Research Center of Mechanical Testing Technology and Equipment (Ministry of Education), Chongqing University of Technology, Chongqing 400054, China; xxj@stu.cqut.edu.cn

**Keywords:** capacitive angle sensors, frequency-domain analysis, frequency drift, random error, systematic error

## Abstract

**Highlights:**

**What are the main findings?**

- Capacitive angle sensors have an optimal operating frequency.
- Operating frequency for capacitive angle sensors does not impact the systematic error, but it does impact the random error and the sensitivity of the angle drift to frequency drift.

**What are the implications of the main findings?**

- Optimal operating frequency of capacitive angle sensors can be determined by locating the point on the amplitude–frequency response curve where the output signal amplitude is maximized.
- The frequency-domain characteristics presented in this paper refine our understanding of capacitive angle sensors and provide guidance for sensor design and optimization.

**Abstract:**

Capacitive angle sensors are crucial components in many industrial applications due to their ability to provide precise angle measurement at low cost and low power consumption. Unfortunately, their frequency-domain characteristics have yet to be thoroughly investigated. In this paper, we investigate the frequency response curves of capacitive angle sensors and find that these sensors have an optimal operating frequency. We also show that operating frequency for capacitive angle sensors does not impact systematic error, but it does impact random error. Based on our findings, we demonstrate the optimization of a candidate sensor’s operating frequency and show a 11.5% ± 1.5% (p = 95% and k = 2.14) reduction in the random error compared to the sensor operating at the typical 20 kHz frequency. The frequency-domain characteristics presented in this paper refine our understanding of capacitive angle sensors and provide guidance for sensor design and optimization.

## 1. Introduction

Angle is a crucial derived quantity in the International System of Units (SI), and angle sensors are fundamental measurement components in numerous industries, such as metrology, manufacturing, automotive, aircraft, and robotics [1,2,3,4]. Angle sensors are typically categorized into three types according to their operating principles: optical encoders, electromagnetic angle sensors, and capacitive angle sensors [5,6]. Optical encoders feature high precision and high resolution, but they are expensive and susceptible to performance degradation and damage in harsh environments [7,8,9]. Therefore, optical encoders are mainly used in metrology laboratories and factories where the environment is well controlled [10]. Compared to optical encoders, electromagnetic angle sensors demonstrate superior performance in harsh environments, but they are bulky, heavy, and power-intensive [11,12]. As a result, electromagnetic angle sensors are typically used in large equipment, such as military vehicles and large ships [13]. Capacitive angle sensors achieve a balance between precision and environmental resilience, and these sensors are typically inexpensive, compact, lightweight, and low-power [6,14,15]. Therefore, capacitive angle sensors are an attractive solution for robotics and drone applications, garnering increased attention in recent years.

Due to the advantages provided by capacitive angle sensors, researchers have developed many miniaturized sensor variants using printed circuit board (PCB) technology for applications with weight and size constraints [16,17,18]. Researchers have also utilized microelectromechanical systems (MEMS) technology to reduce size and power consumption beyond what is achievable with PCB-based sensors [15,19,20]. In certain applications where space is a limiting factor, three-plate, cylindrical, and multi-layer ring structures provide enhanced coupling capacitance in a small form factor [21,22,23,24,25]. With regard to sensor signal quality, the optimized angle demodulation circuits have been used to enhance anti-interference capabilities and reduce costs [26,27,28]. The error compensation techniques have been introduced to enhance the accuracy of capacitive angle sensors [19,29]. The studies outlined above examine various aspects of capacitive angle sensors, including utilization, manufacturing, cost, structure, interference resistance, resolution, and accuracy. However, frequency response of capacitive angle sensors has not been investigated, and the mechanism by which operating frequency affects sensor performance remains unclear. As a result, no guidance exists in the selection of operating frequency for capacitive angle sensors.

In this paper, we investigate the frequency response curves of a typical capacitive angle sensor at different rotation angles to address the lack of research in frequency response. We investigate the effects of operating frequency on the sensor’s random error, frequency drift, resolution, and systematic error. Moreover, we characterize the underlying mechanisms of these effects. Our results indicate that systematic error is minimally impacted by the operating frequency, but the random error, resolution, and the sensitivity of angle drift to frequency drift are significantly impacted by the operating frequency. Based on the mechanisms affecting these performance aspects, we present an approach to optimize the sensor’s operating frequency, and our experimental results show a random error reduction of 10% with no change in systematic error. The frequency-domain analysis results presented in this paper refine our understanding of capacitive angle sensors and provide guidance for future research and design optimization.

## 2. Capacitive Angle Sensor

Capacitive angle sensors use the change in common plate area between two electrodes to modulate the angle into an electrical signal, and similar electrode geometries, circuits, and components are used in most capacitive angle sensors [6,14,17,20]. Therefore, we present a representative capacitive angle sensor using common design methods to analyze its frequency-domain characteristics.

### 2.1. Measurement Principle

**Figure 1:**
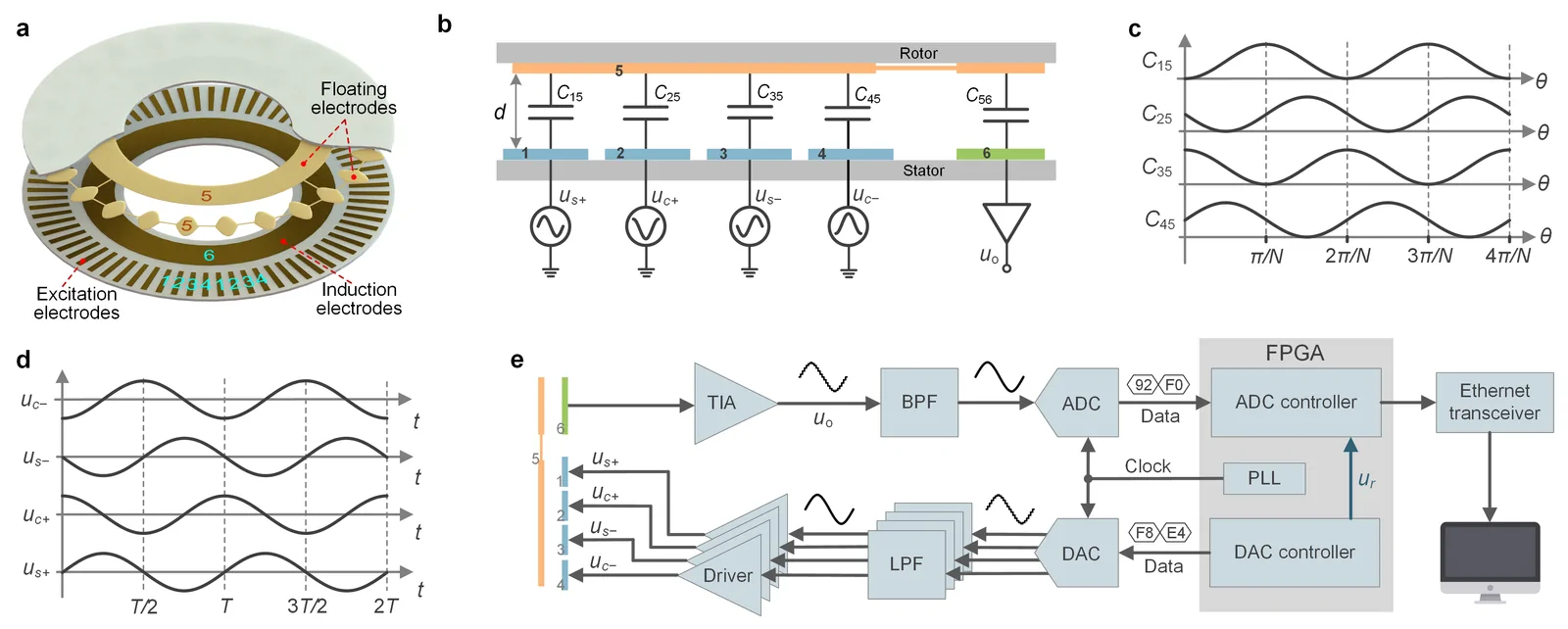
Representative capacitive angle sensor: (**a**) structural diagram of the sensor; (**b**) sensing capacitance and induction capacitor of the sensor; (**c**) ideal sensing capacitance curves; (**d**) excitation voltage signals; (**e**) block diagram of the sensor signal processing circuit.

The geometric structure of the capacitive angle sensor is shown in Figure 1a. The excitation and induction electrodes are mounted on the stator, and the floating electrode is mounted on the rotor. The excitation electrodes are divided into groups, labeled 1, 2, 3, and 4. The floating electrode and the induction electrode are labeled 5 and 6, respectively. The sensing capacitors (*C*_15_, *C*_25_, *C*_35_, *C*_45_) are created between the sector-shaped excitation electrodes and the sinusoidal-shaped floating electrodes when the stator and rotor are mounted with an air gap, as shown in Figure 1b. When the rotor rotates, the common plate areas between excitation electrodes 1, 2, 3, and 4 and the floating electrode can be expressed as [30]

(1) $$\left\{\begin{array}{l} S_{15}=\left(S_{m}/2\right)\left[1-\cos\left(N\theta \right)\right]\\ S_{25}=\left(S_{m}/2\right)\left[1-\sin\left(N\theta \right)\right]\\ S_{35}=\left(S_{m}/2\right)\left[1+\cos\left(N\theta \right)\right]\\ S_{45}=\left(S_{m}/2\right)\left[1+\sin\left(N\theta \right)\right] \end{array}\right.$$

where *S_m_*, *N*, and *θ* represent the maximum common plate area, number of mechanical cycles of the angle sensor, and the rotation angle, respectively. If the fringe effect is neglected, the sensing capacitances between the excitation electrodes and the floating electrode can then be expressed as

(2) $$\left\{\begin{array}{l} C_{15}=\left(\varepsilon S_{m}/2d\right)\left[1-\cos\left(N\theta \right)\right]\\ C_{25}=\left(\varepsilon S_{m}/2d\right)\left[1-\sin\left(N\theta \right)\right]\\ C_{35}=\left(\varepsilon S_{m}/2d\right)\left[1+\cos\left(N\theta \right)\right]\\ C_{45}=\left(\varepsilon S_{m}/2d\right)\left[1+\sin\left(N\theta \right)\right] \end{array}\right.,$$

where *ε* represents the dielectric constant of air, and d represents the separation distance, or gap, between the excitation and floating electrodes. The sensing capacitance values change with the rotation angle, as shown in Figure 1c.

To measure the rotation angle, four excitation voltage signals, each phase-shifted by 90° relative to the preceding one, are connected to four sets of excitation electrodes. These four excitation voltage signals can be represented as

(3) $$\left\{\begin{array}{l} u_{s+}=A_{m}\sin\left(2\pi ft\right)\\ u_{c+}=A_{m}\cos\left(2\pi ft\right)\\ u_{s-}=-A_{m}\sin\left(2\pi ft\right)\\ u_{c-}=-A_{m}\cos\left(2\pi ft\right) \end{array}\right.,$$

where *A_m_*, *f*, and *t* indicate the amplitude, frequency, and time of the excitation voltage signals, respectively. The excitation voltage varies with time, as shown in Figure 1d, and the four excitation voltages will be superimposed on electrode 5 after being coupled through the sensing capacitors. According to superposition principle, we can calculate the voltage signal *u*_5_ at electrode 5 as

(4) $$u_{5}=\frac{C_{15}u_{s+}+C_{25}u_{c+}+C_{35}u_{s-}+C_{45}u_{c-}}{C_{15}+C_{25}+C_{35}+C_{45}+C_{56}},$$

where *C*_56_ is the induction capacitor between floating electrode 5 and induction electrode 6. Using (2) and (3), (4) can be rewritten as [31]

(5) $$u_{5}=-K\sin\left(2\pi ft+\varphi \right),$$

where *K* = *εS_m_A_m_*/[*d*(*C*_15_ + *C*_25_ + *C*_35_ + *C*_45_ + *C*_56_)], representing the amplitude of *u*_5_, and *φ* = *Nθ*, representing the initial phase of *u*_5_. The output signal, *u*_o_, can be expressed as

(6) $$u_{o}=\frac{K\cdot C_{56}}{C_{f}+1/\left(2j\pi f\cdot R_{f}\right)}\sin\left(2\pi ft+\varphi \right),$$

where *C_f_* and *R_f_* represent the feedback capacitance and feedback resistance of the trans-impedance amplifier (TIA), respectively. *θ* is linearly related to *φ*, and as long as we measure the initial phase *φ* of *u*_5_, *θ* is given by

(7) $$\theta =\frac{\varphi }{N}.$$

### 2.2. Signal Processing

A block diagram of the sensor signal processing circuit is shown in Figure 1e. We use a field-programmable gate array (FPGA) as the core processor to control the analog-to-digital converter (ADC), digital-to-analog converter (DAC), and Ethernet transmitter. The DAC generates four excitation voltage signals (*u_s_*_+_, *u_c_*_+_, *u_s_*_−_, *u_c_*_−_), and low-pass filters (LPFs) remove high-frequency ripple from the excitation signals. *u*_r_ is the trigger signal for the ADC. The drivers enhance the system’s ability to drive capacitive loads, and the excitation voltage signals are applied to the excitation electrodes through the drivers. The signal *u*_5_ at electrode 5 is amplified to an output voltage signal, *u_o_*, by the induction capacitor *C*_56_ and the TIA, and a band-pass filter (BPF) suppresses interference and noise in *u_o_*. We set the BPF’s passband to 500 Hz–2 MHz. The ADC converts the filtered output voltage signal into binary data that is sent to a computer through the Ethernet transmitter. We employ the computer to calculate the initial phase, *φ*, of *u_o_* using a sine-fitting algorithm [32], and obtain *θ* using (7).

### 2.3. Prototype Fabrication and Experimental Setup

**Figure 2:**
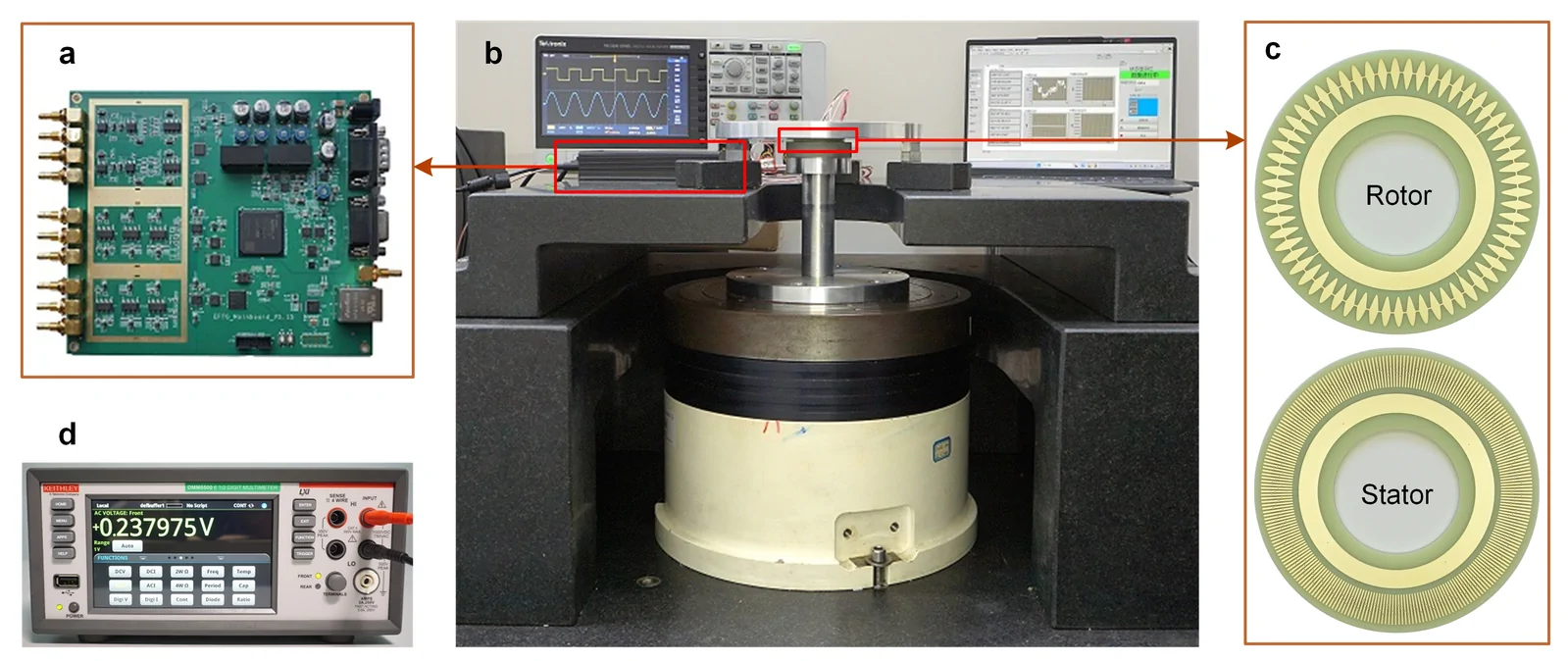
Experimental setup: (**a**) signal processing circuit board for the capacitive angle sensor; (**b**) complete experimental assembly; (**c**) stator and rotor for the capacitive angle sensor; (**d**) signal calibration.

To analyze the frequency-domain characteristics of the capacitive angle sensor, we fabricated a prototype sensor and configured the experimental system, as shown in Figure 2. We fabricated the stator and rotor of the sensor on PCBs with a mechanical cycle of 60, as shown in Figure 2c. We mounted the stator on a height-adjustable bracket and the rotor on an AP 300 high-precision turntable (RPI, Bath, UK), as shown in Figure 2b. We also fabricated the signal processing circuit described in Section 2.2, as shown in Figure 2a. The RON905 optical encoder (Heidenhain, Traunreut, Germany) embedded in the turntable is used to evaluate the error of capacitive angle sensor; it has an accuracy of ±0.4″ as an angle comparator. We used a PXIe-5122 high-resolution oscilloscope (NI, Austin, USA), a 6000A phase meter (Clarke-Hess, New York, NY, USA), and a DMM6500 digital multimeter (Tektronix, Beaverton, OR, USA) to measure the signal amplitude and phase and calibrate the signal processing circuit. We also precisely controlled the laboratory environment, maintaining a stable temperature of 20 ± 1 °C and a relative humidity of 50 ± 1%. Table 1 provides a detailed list of our test setup specifications.

## 3. Frequency-Domain Analysis

### 3.1. Qualitative Frequency-Domain Analysis

**Figure 3:**
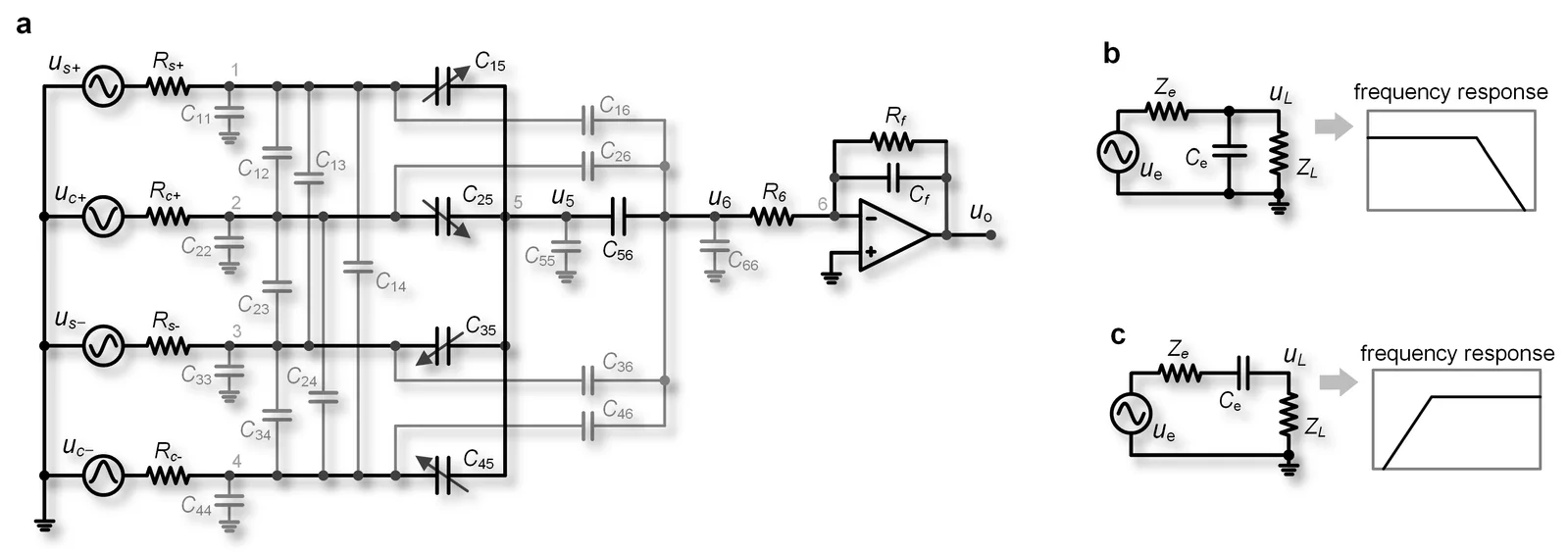
Circuit models and qualitative analysis: (**a**) a basic circuit model for the capacitive angle sensor; (**b**) frequency response of a parallel capacitor; (**c**) frequency response of a series capacitor.

The best mathematical tool for frequency-domain analysis is the transfer function of a circuit. Accordingly, we attempt to establish a basic circuit model for the capacitive angle sensor to obtain the sensor’s transfer function. This basic circuit model contains four excitation voltage sources (*u_s_*_+_, *u_c_*_+_, *u_s_*_−_, *u_c_*_−_), four sensing capacitors (*C*_15_, *C*_25_, *C*_35_, *C*_45_), one induction capacitor (*C*_56_), 16 parasitic capacitors (*C*_11_, *C*_12_, *C*_13_, *C*_14_, *C*_16_, *C*_22_, *C*_23_, *C*_24_, *C*_26_, *C*_33_, *C*_34_, *C*_36_, *C*_44_, *C*_46_, *C*_55_, *C*_66_), cable resistances (*R_s_*_+_, *R_c_*_+_, *R_s_*_−_, *R*_c−_, *R*_6_), one feedback resistor (*R*_f_), one feedback capacitor (*C*_f_), and one operational amplifier, as shown in Figure 3a. We model the capacitive angle sensor as a complex capacitive network consisting of 21 capacitors in the basic circuit model, but the transfer function of the circuit model is very difficult to obtain due to the complexity of the capacitive network [15]. Since the parasitic capacitance is comparable in magnitude to the sensing capacitance, it is also very difficult to simplify the circuit model. Moreover, the capacitance values are also difficult to measure because of signal cross-coupling within the network. Therefore, theoretical frequency domain analysis of capacitive angle sensors presents considerable challenges. Nevertheless, we can qualitatively analyze the frequency-domain characteristics of capacitive angle sensors. The capacitive reactance of a capacitor decreases as the signal frequency increases. When a capacitor is connected in parallel with the load impedance, high-frequency signals are attenuated, as shown in Figure 3b. When a capacitor is connected in series with the load impedance, low-frequency signals are attenuated, as shown in Figure 3c. The basic circuit model of a capacitive angle sensor includes multiple capacitors connected in parallel and in series, as shown in Figure 3a. Therefore, both high-frequency and low-frequency signals are attenuated as they pass through the capacitive angle sensor. This characteristic may raise new considerations regarding the selection of operating frequency for capacitive angle sensors. Thus, we attempt to analyze the sensor’s frequency-domain characteristics in experiments by measuring the amplitude and phase of the output signal at different frequencies. Note that the subsequent frequency-domain analysis and optimization are established experimentally rather than theoretically.

### 3.2. Amplitude–Frequency Characteristics

**Figure 4:**
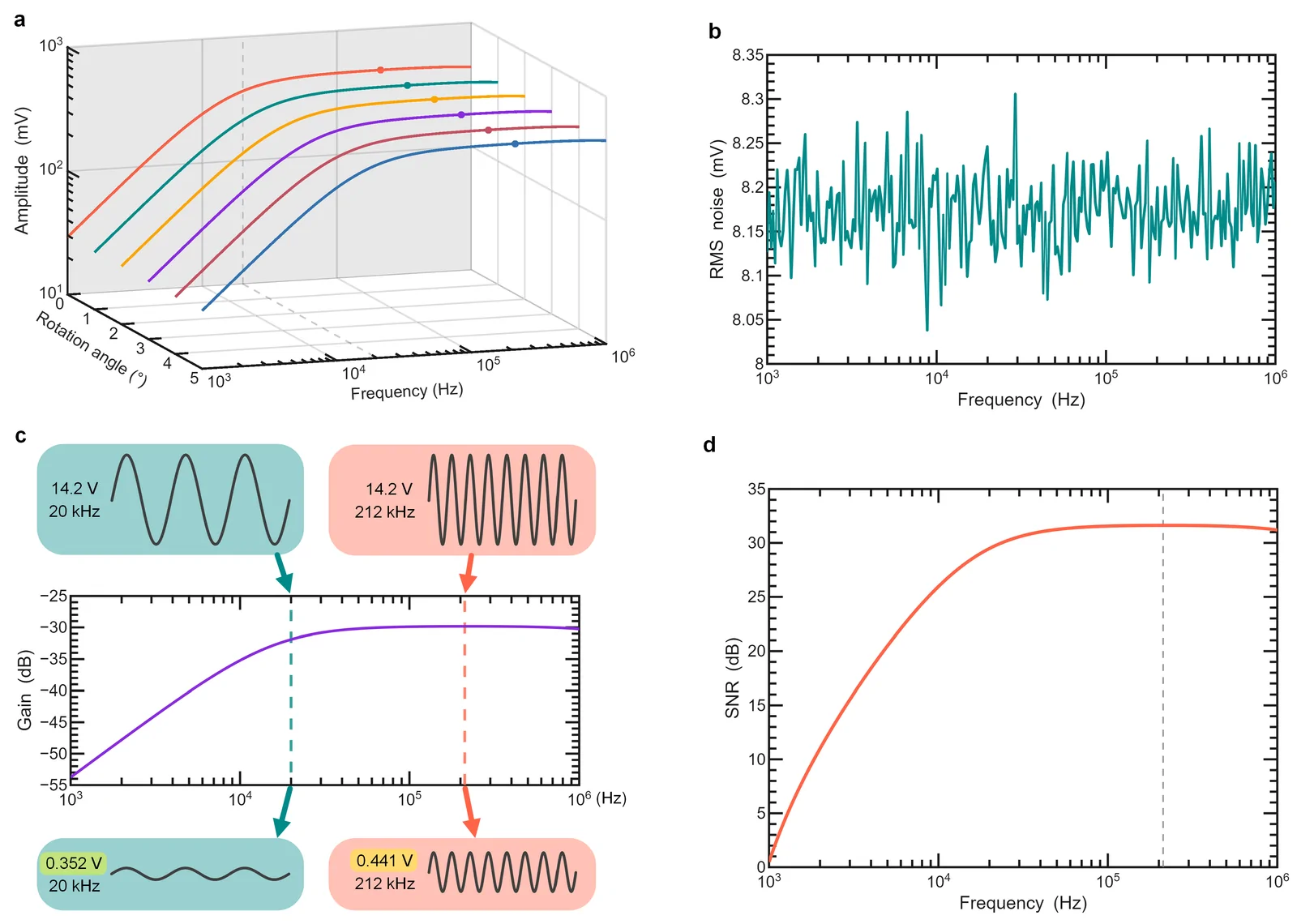
Amplitude–frequency analysis results for the capacitive angle sensor: (**a**) amplitude–frequency response curves at different rotation angles; (**b**) RMS noise as a function of operating frequency; (**c**) amplitude and gain characteristics at different operating frequencies; (**d**) SNR as a function of operating frequency.

To perform the frequency-domain analysis, we set 256 frequency points in the range from 1 kHz to 1 MHz and measured the amplitude of *u*_o_ to obtain the amplitude–frequency response at different rotation angles, as shown in Figure 4a. Within the range of 0° to 360°, the frequency response curves are nearly identical at each angle. Only the frequency response curves from 0° to 5° are shown, as these curves repeat periodically in accordance with the mechanical cycle (6°). We observed that maximum amplitude occurred at 212 kHz, and the point of maximum amplitude is marked with solid small dots on each amplitude–frequency response curve, as shown in Figure 4a. The signal amplitude gradually decreases as the operating frequency decreases from 212 kHz. Meanwhile, the signal amplitude gradually decreases as the operating frequency increases from 212 kHz. This result confirms the existence of the frequency with maximum amplitude. The described amplitude–frequency characteristics are a combination of the sensor’s intrinsic characteristics and the TIA’s characteristics.

The operating frequency of 20 kHz is widely adopted as the operating frequency for capacitive angle sensors [17,18,19,31,32,33]. However, 20 kHz is not the optimal frequency according to the amplitude–frequency response curve. Generally, we want the sensor to operate at the frequency where the gain is highest to achieve the best signal-to-noise ratio (SNR). We measured the noise voltage 10 times at each frequency point. The RMS noise voltage at the 256 frequency points measured during the first test is plotted in Figure 4b. The noise at each frequency point was randomly distributed around 8.1764 mV. The results of the significance test showed that *p* = 0.12 > 0.05, and no significant differences were found among the 256 frequency points (10 noise voltage values per frequency point). Therefore, the experimental results show that the amplitude of the noise remains constant. Capacitive angle sensors exhibit different gains for signals of different frequencies, as shown in Figure 4c. The sensor operates at a single frequency, but noise is generally distributed over a very wide frequency band. When the sensor’s operating frequency changes, the amplitude of the output signal changes, but the amplitude of the noise remains constant, causing changes in the output SNR.

The relationship between SNR and frequency is shown in Figure 4d. According to our experimental results, when the sensor operated at a frequency of 212 kHz, the output signal amplitude reached a maximum of 0.441 V (RMS value of 311.8 mV), with an SNR of 31.629 ± 0.141 dB (*p* = 99% and *k* = 3.25). However, the output signal amplitude was only 0.352 V (RMS value of 248.9 mV), and the SNR was 29.672 ± 0.141 dB (*p* = 99% and *k* = 3.25) with an operating frequency of 20 kHz. Therefore, the optimal SNR was achieved when the operating frequency was 212 kHz. The SNR maintained a high level around 212 kHz, as shown in Figure 4d. If the goal is not to achieve the highest SNR, the operating frequency can also be set to around 212 kHz. Note that since the sinusoidal voltage signal serves as the carrier for angle modulation and demodulation, the SNR is defined by the ratio of voltages, not power. As with most capacitive sensors, the SNR of the output signal is the primary factor determining the sensor’s random error [34,35]. The maximum signal amplitude results in the optimal SNR, which in turn leads to the optimal random error. Therefore, the amplitude–frequency characteristics shown in Figure 4a provide a basis for selecting the optimal operating frequency to maximize SNR and optimize the sensor’s random error.

### 3.3. Phase–Frequency Characteristics

**Figure 5:**
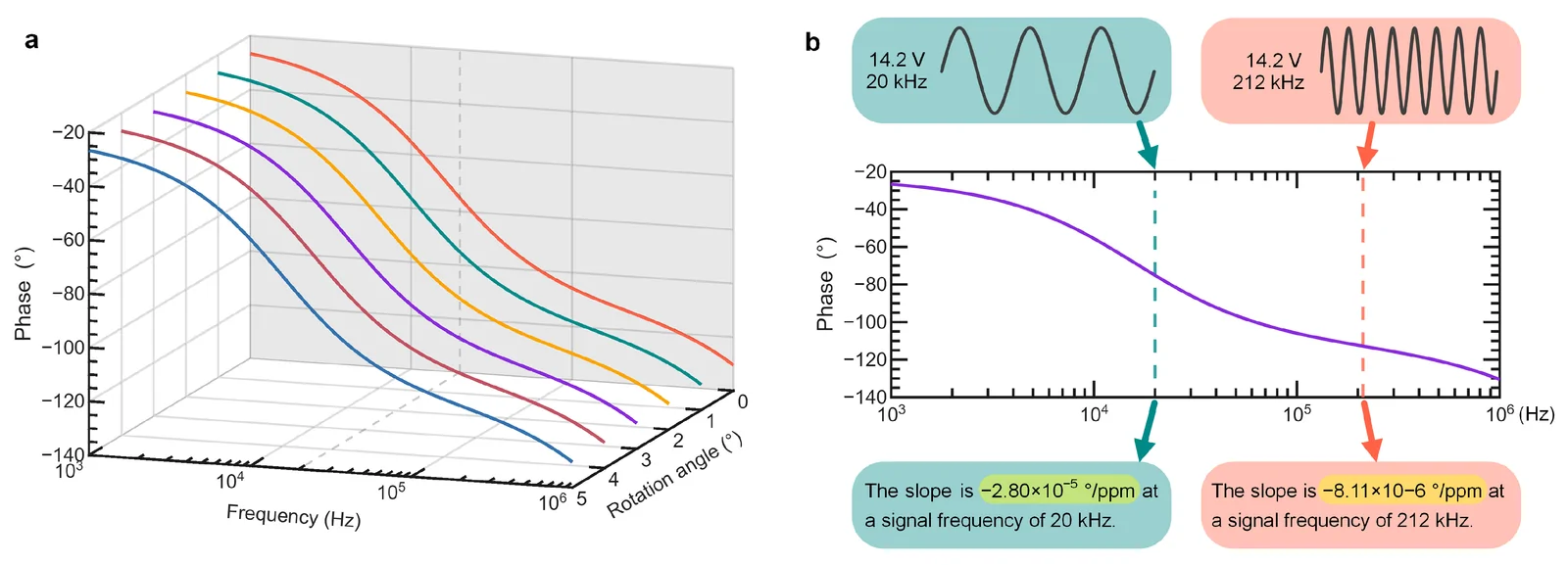
Phase–frequency analysis results for the capacitive angle sensor: (**a**) phase–frequency response curves at different rotation angles; (**b**) phase and its slope characteristics at different operating frequencies.

Our experimental phase–frequency response is shown in Figure 5a. Notably, the phase shifts linearly as the rotor rotates. However, we removed the phase shift caused by rotation in order to investigate only the phase shift caused by frequency and to achieve clearer phase–frequency characteristics. These curves indicate consistent phase–frequency characteristics at different rotation angles. Only the frequency response curves from 0° to 5° are shown, as these curves repeat periodically in accordance with the mechanical cycle (6°).

The phase–frequency response curve illustrates the relationship between phase drift and frequency drift. The rotation angle exhibits a linear relationship with the phase of the output signal according to (7). The sensitivity *k_θ_* of angle drift to frequency drift can be expressed as

(8) $$k_{\theta }=\frac{1}{N}\cdot \frac{d\varphi }{df};$$

here, (*dφ*)/(*df*) is the slope of the phase–frequency response curve. A steeper slope in the phase–frequency response curve indicates greater phase drift caused by a unit frequency drift, resulting in a larger angle drift (Figure 5b). According to our experimental results, the slopes of the phase–frequency response curve were −2.80 × 10^−5^ °/ppm and −8.11 × 10^−6^ °/ppm at operating frequencies of 20 kHz and 212 kHz, respectively. Furthermore, we observed that the slope was smallest at 212 kHz. Therefore, the angle drift caused by frequency drift was minimal with an operating frequency of 212 kHz. Phase–frequency characteristics provide a basis for optimizing the sensitivity of angle drift to frequency drift. Both the phase–frequency and amplitude–frequency analysis results indicate that 212 kHz is the optimal operating frequency for the proposed sensor.

### 3.4. Side Effects of Increasing Operating Frequency

The above analysis suggests increasing the frequency to 212 kHz, but the theoretical resolution of capacitive angle sensors declines as the operating frequency increases. Theoretical resolution, *R_θ_*, is determined by the number of mechanical cycles, *N*, the operating frequency, *f*, the ADC sampling frequency, *f_s_*, and the number of quantization bits, *Q*, which can be expressed as [32]

(9) $$R_{\theta }=360^{{}^{\circ}}\frac{f}{2^{Q}Nf_{s}}.$$

For operating frequencies of 20 kHz and 212 kHz, the theoretical resolutions are 3.96 × 10^−4^″ and 4.20 × 10^−3^″, respectively. Therefore, the theoretical resolution declines by a factor of 10.6.

## 4. Optimization Results

### 4.1. Random Error

**Figure 6:**
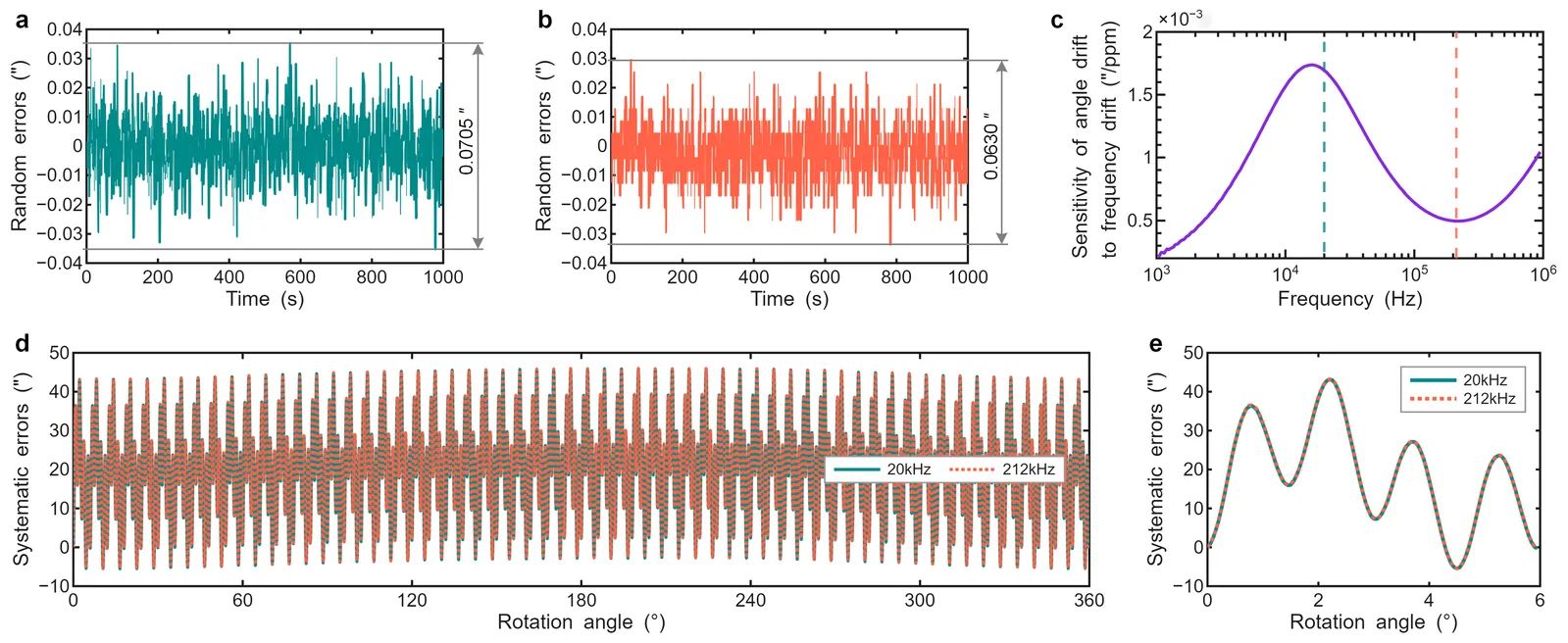
Experimental frequency optimization results: (**a**) sensor random errors before frequency optimization (20 kHz); (**b**) sensor random errors after frequency optimization (212 kHz); (**c**) sensitivity of angle drift to frequency drift at different operating frequencies; (**d**) angle errors over the full 0–360° range at frequencies of 20 kHz and 212 kHz; (**e**) angle error within the 0–6° range at frequencies of 20 kHz and 212 kHz.

Random error is defined as the peak-to-peak value of the angle jitter measured when the rotor is stationary. To verify the results of the amplitude–frequency analysis, we monitored the sensor’s rotation angle during the experiment while the rotor was stationary. We recorded a set of random errors every hour on a prototype. A set of random errors contains 1000 samples, with a sampling duration of 1000 s and a sampling rate of 1 Hz. Table 2 shows the statistical results for eight sets of random error data recorded within an 8-h period. The mean value of eight sets of random error data was adopted as the experimental result. When the operating frequency was 20 kHz, the peak-to-peak value of the random errors was 0.0702″, with a standard deviation of 0.0107″, as shown in Table 2. When the operating frequency was 212 kHz, the peak-to-peak value of the random errors was 0.0627″, with a standard deviation of 0.0095″, as shown in Table 2. The significance test indicated that the difference in random error between 20 kHz and 212 kHz was highly statistically significant. Therefore, the random error of the capacitive angle sensor was decreased by 11.5% ± 1.5% (*p* = 95% and k = 2.14) by changing the operating frequency to 212 kHz. Since the noise from the mechanical/turntable did not vary with the SNR, an approximately 25% improvement in the SNR resulted in only an 11.5% reduction in random error. To illustrate the optimization results more clearly, we present a set of random error data before and after optimization in Figure 6a and Figure 6b, respectively. The random error was successfully optimized by selecting the optimal operating frequency based on the sensor’s amplitude–frequency characteristics. The optimal frequency of 212 kHz applies only to specific sensors (our prototype), but the method for determining the optimal frequency by locating the amplitude peak is generally applicable.

When the theoretical resolution falls below the random error, the sensor’s random error becomes the dominant factor influencing its performance. The theoretical resolution of 0.0042″ was significantly smaller than the random error of 0.0627″ at 212 kHz. Therefore, it is well worth sacrificing theoretical resolution in exchange for decreased random error.

### 4.2. Sensitivity of Angle Drift to Frequency Drift

Based on the experimental results, the sensitivity curve of the angle drift to frequency drift is plotted in Figure 6c. The maximum angle drift sensitivity was 1.68 × 10^−3^″/ppm at an operating frequency of 20 kHz, and the minimum was 4.87 × 10^−4^″/ppm at an operating frequency of 212 kHz. The frequency stability of the crystal oscillator was 2.5 ppm in the proposed sensor. When the operating frequency was optimized from 20 kHz to 212 kHz, the value of angle drift caused by frequency drift was optimized from 4.2 × 10^−3^″ to 1.2 × 10^−3^″, and the sensitivity of the angle drift to frequency drift decreased by 72%. These values of angle drift caused by frequency drift are negligible compared to the systematic error of the capacitive angle sensor, as shown by the data in Table 3. These results demonstrate that the sensor’s error is scarcely affected by slight frequency drift, which improves our understanding of the errors in capacitive angle sensors.

### 4.3. Systematic Error

We recorded the rotation angle values of the capacitive angle sensor within the range of 0° to 360°, and we determined sensor systematic errors by comparing these angle values with the reference angle values output by the high-precision optical encoder. The systematic error curves measured at 20 kHz and 212 kHz are shown in Figure 6d, and the systematic error curves for one mechanical cycle are shown in Figure 6e. The systematic error was ±25.8″ within the range of 0° to 360°. Moreover, we observed strong agreement between the data at 20 kHz and 212 kHz, as shown in Figure 6d,e. Within the range of 0° to 360°, the maximum deviation between these two sets of data was only 0.14″, and the system error at 212 kHz is smaller than at 20 kHz. This deviation of 0.14″ is smaller than the accuracy of the turntable and the optical encoder, and far smaller than the error of the capacitive angle sensor. Therefore, no significant influence of operating frequency on systematic error was observed under the current conditions.

A comparison of different capacitive angle sensors is provided in Table 3. The sensors reported in references [27,28] exhibit suboptimal performance because they were designed to achieve extremely low costs. The systematic error of the proposed sensor is not the lowest, but its resolution and random error are significantly better than those of existing capacitive angle sensors. The high resolution and low random error achieved in this work establish a baseline for expanding the applications of capacitive angle sensors.

## 5. Discussion

In this paper, we experimentally investigated the frequency-domain characteristics of capacitive angle sensors, and we present the following primary conclusions based on our analysis:

1. There is an optimal operating frequency for capacitive angle sensors.
2. We can determine the optimal operating frequency of capacitive angle sensors by locating the point on the amplitude–frequency response curve where the output signal amplitude is maximized.
3. The choice of operating frequency for capacitive angle sensors does not impact the systematic error, but it does impact the random error and the sensitivity of the angle drift to frequency drift.

In addition to developing these general conclusions, we described the physical mechanisms by which operating frequency affects random error and sensitivity of the angle drift to frequency drift. We also reduced the random error of the candidate sensor based on the results of the frequency-domain analysis, but the theoretical resolution was compromised. Therefore, it is necessary to balance random error and resolution for sensors exhibiting lower resolution.

The work presented in this paper advances our understanding of capacitive angle sensors and provides valuable insight for optimizing operating frequency. Our analytical methods and conclusions can serve as a reference for other capacitive sensors. Besides operating frequency, other characteristics of capacitive angle sensors remain to be thoroughly investigated, such as electrode geometry, assembly precision, operating temperature, and operating humidity. We intend to investigate these parameters in future work.

## Figures and Tables

**Table 1:** Test setup specifications.

Symbol	Parameters	Value
-	Outer diameter	45 mm
-	Inner diameter	20 mm
*d*	Air gap between stator and rotor	0.3 mm
-	Number of excitation electrodes	240
-	Number of floating electrodes	60
*N*	Number of mechanical cycles	60
*A_m_*	Amplitude of excitation voltage	14.2 V
*f*	Frequency of excitation voltage	1–1000 kHz
*R_f_*	Feedback resistor	1 MΩ
*C_f_*	Feedback capacitor	10 pF
*f_s_*	ADC sampling frequency	66.667 MHz
*Q*	ADC quantization bits	14
-	Noise/measurement bandwidth	20 MHz

**Table 2:** Random error from eight measurements.

Frequency	Statistical Results	Random Errors (″)								Mean (″)
20 kHz	Peak-to-peak value	0.0703	0.0701	0.0705	0.0702	0.0703	0.0700	0.0699	0.0702	0.0702
	Standard deviation	0.0108	0.0106	0.0110	0.0107	0.0107	0.0106	0.0105	0.0107	0.0107
212 kHz	Peak-to-peak value	0.0628	0.0626	0.0630	0.0627	0.0629	0.0625	0.0624	0.0626	0.0627
	Standard deviation	0.0095	0.0094	0.0097	0.0095	0.0096	0.0093	0.0093	0.0094	0.0095

**Table 3:** Comparison of different capacitive angle sensors.

From	[17]	[18]	[19]	[20]	[27]	[28]	[31]	[33]	This Work
Diameter	57 mm	54 mm	58 mm	27.5 mm	29.18 mm	-	60 mm	83 mm	45 mm
Frequency	20 kHz	20 kHz	≤20 kHz	-	6 kHz	250 kHz and 500 kHz	20 kHz	20 kHz	212 kHz
Random error	2″	2.16″	0.36″	7.2″	720″	330″	2.5″	0.5″	0.063″
Resolution	0.2″	0.54″	0.72″ *	7.2″ *	72″ *	22.68″ *	0.23″	0.18″	0.0042″
Systematic error	±25″	±4″	4.32″	86.4″	3456″	3758″	±10″	12″	±25.8″

* Values marked with an asterisk (*) represent measured resolutions and cannot be directly compared with other theoretical resolutions.

## Data Availability

The original contributions presented in this study are included in the article. Further inquiries can be directed to the corresponding author.
